# LncRNA PACER is down-regulated in osteoarthritis and regulates chondrocyte apoptosis and lncRNA HOTAIR expression

**DOI:** 10.1042/BSR20190404

**Published:** 2019-06-07

**Authors:** Mingwei Jiang, Jie Liu, Tao Luo, Qiu Chen, Ming Lu, Deqiang Meng

**Affiliations:** 1 Department of orthopaedics, Shanghai Public Health Clinical Center, Shanghai City 201508, P.R. China; 2 Department of orthopaedics, Hangzhou First People’s Hospital, Hangzhou City, Zhejiang Province 310006, P.R. China

**Keywords:** chondrocyte, lncRNA PACER, lncRNA HOTAIR, osteoarthritis

## Abstract

LncRNA PACER is a chondrocyte inflammation-associated long non-coding RNA (lncRNA), and chondrocyte inflammation is involved in osteoarthritis (OA). We observed that plasma PACER was down-regulated, while plasma HOTAIR was up-regulated in OA patients. Altered plasma levels of PACER and HOTAIR distinguished OA patients from healthy controls. PACER and HOTAIR were inversely correlated in both OA patients and healthy controls. PACER overexpression mediated the down-regulation of HOTAIR, while HOTAIR overexpression did not significantly affect PACER. PACER overexpression led to inhibited, while HOTAIR overexpression led to promoted apoptosis of chondrocyte. HOTAIR overexpression attenuated the effects of PACER overexpression. Therefore, lncRNA PACER is down-regulated in OA and regulates chondrocyte apoptosis by down-regulating lncRNA HOTAIR.

## Introduction

Osteoarthritis (OA) is a prevalent joint degenerative disease that is characterized by the loss of cartilage and the formation of new bone on joint surface, which lead to physical disability [1]. Osteoarthritis is a major cause of and disability pain. In effect, degeneration of medial compartment is inevitable during the development of OA [2]. OA mainly affects patients older than 50 years, and the incidence is even higher among the population >65 years old [3]. At present, OA has become a major public health problem [4]. End-stage OA can be treated by joint replacement, while the short life span of prostheses and poor functional outcomes limit the clinical application of this technique [2–4]. Previous studies have characterized multiple risk factors for OA. Those risk factors may include aging, overweight and obese [5]. However, pathogenesis of this disease remains unclear, leading to the difficulties in clinical treatment.

Long (>200 nt) non-coding RNAs (lncRNAs) are emerging classes of non-protein coding RNA transcripts with critical functions in both physiological pathological processes [6,7]. Previous studies also provided evidence that lncRNAs also participate in inflammatory responses [8], which contribute to the development and progression of OA [9,10]. In effect, certain lncRNAs can participate in OA by regulating related signaling pathways and cell behaviors [11]. For instance, HOTAIR is up-regulated in OA and participates in IL-1β-induced MMP overexpression and promotes the apoptosis of chondrocytes [11]. LncRNA PACER has been characterized as a chondrocyte inflammation–associated lncRNA and chondrocyte inflammation contributes to OA [12], indicating its potential involvement in OA. Our preliminary RNA-seq experiment revealed the altered expression of PACER in OA patients and its inverse correlation with HOTAIR. Our study was therefore carried out to explore the possible involvement of PACER in OA and to investigate its potential interaction with HOTAIR.

## Materials and methods

### Subjects

A total of 73 patients with OA (patient group) and 66 healthy volunteers (control group) were enrolled in Shanghai Public Health Clinical Center from January 2016 to January 2018. Magnetic resonance imaging and joint fluid analysis were used to diagnose the disease. Patients inclusion criteria: (1) first time diagnosis; (2) no medicine taken within 3 months. Patients’ exclusion criteria: (1) other chronic diseases were observed; (2) recurrent cases. The 73 patients included 30 males and 43 females, and age ranged from 47 to 71 years old, with a mean of 58.7 ± 5.2 years. The 66 healthy volunteers were enrolled at physical health center. All healthy controls showed physiological parameters within normal range. The 66 healthy volunteers included 26 males and 40 females, and age ranged from 46 to 73 years old, with a mean of 58.3 ± 6.1 years. Informed consent was provided by all patients. The present study was approved by Ethics Committee of aforementioned institute before the admission of participants. The research has been carried out in accordance with the World Medical Association Declaration of Helsinki.

### Plasma specimens and cell line

Before the initiation of therapies, fasting blood (5 ml) was extracted and centrifuged (1200 ***g***) in EDTA-treated tubes to separate plasma.

CHON-001 human chondrocyte cell line (ATCC, U.S.A.) was used. Cells were cultivated in Dulbecco’s Modified Eagle’s Medium (10% FBS and 0.1mg/ml G-418) in an incubator (37°C, 5% CO_2_).

### RT-qPCR

Trizol reagent (Invitrogen, U.S.A.) was directly mixed with CHON-001 cells or plasma to perform total RNA extractions. All RNA samples were digested with DNase I (Sangon, Shanghai, China) for 1 h at 37°C before use. SuperScript III Reverse Transcriptase (Thermo Fisher Scientific., lnc.) was used to transcribe total RNAs to prepare cDNA. To detect PACER and HOTAIR, Applied Biosystems™ PowerUp™ SYBR™ Green Master Mix was used to prepare PCR mixtures with 18S RNA as endogenous control. Reaction conditions were: 2 min at 95°C, followed by 40 cycles of 10 s at 95°C and 40 s at 58°C. Expression of PACER and HOTAIR was normalized to the expression of 18S RNA according to 2^−ΔΔ*C*^_T_ method. Primer sequences were: 5′TGTAAATAGTTAATGTGAGCTCCA3′ (forward) and 5′GCAAATTCTGGCCATCGC3′ (reverse) for PACER; 5′GGTAGAAAAAGCAACCACGAAG3′ (forward) and 5′ACATAAACCTCTGTCTGTGAGTGCC3′ (reverse) for HOTAIR; 5′GCTTAATTTGACTCAACACGGGA3′ (forward) and 5′AGCTATCAATCTGTCAATCCTGTC3′ (reverse) for 18S rRNA. It is worth noting that similar data were obtain using GAPDH as endogenous control.

### Vector construction and cell transfection

Full length PACER and HOTAIR genomic DNAs were inserted into pcDNA3.1 vectors to establish PACER and HOTAIR expression vectors. The vector construction was completed by Sangon (Shanghai, China). Negative control siRNA as well as PACER and HOTAIR siRNAs were from RIBOBIO (Guangzhou, China). 10 nM vectors or 45 nM siRNAs were transfected into 6 × 10^5^ cells of CHON-001 cell line using lipofectamine 2000 reagent (Thermo Fisher Scientific., lnc.). Transfection with empty vectors or negative control siRNA was negative control (NC) and un-transfected cells were control (C) cells, respectively. Cells were harvested at 24 h after transfection to perform subsequent experiments.

### *In vitro* cell apoptosis assay

Cells of CHON-001 cell line were collected at 24 h after transfection to evaluate cell apoptosis through *in vitro* cell apoptosis assay. Briefly, serum-free Dulbecco’s Modified Eagle’s Medium was used to prepare single cell suspensions, and cell density was adjusted to 3 × 10^4^ cells per ml. Each well of a six-well plate was filled with 2 ml cell suspension, followed by cell culture in an incubator (37°C, 5% CO_2_) for 36 h. After that, 0.25% trypsin digestion was performed, followed by staining using Annexin V-FITC and propidium iodide (Dojindo, Japan). Finally, apoptotic cells were detected by flow cytometry.

### Statistical analysis

Mean values of three biological replicates were calculated and processed using Graphpad Prism 6 software. Differences between OA patients and healthy controls were analyzed by unpaired *t*-test. Differences among different cell treatment groups were explored by ANOVA (one-way) and Tukey test. Correlations between expression levels of PACER and HOTAIR were analyzed by linear regression. Diagnostic values of plasma PACER and HOTAIR for OA were analyzed by ROC curve. In ROC curve, true positive cases were OA patients and true negative cases were healthy controls. The *P*<0.05 was statistically significant.

## Results

### Expression levels of PACER and HOTAIR were altered in OA

**Figure 1:**
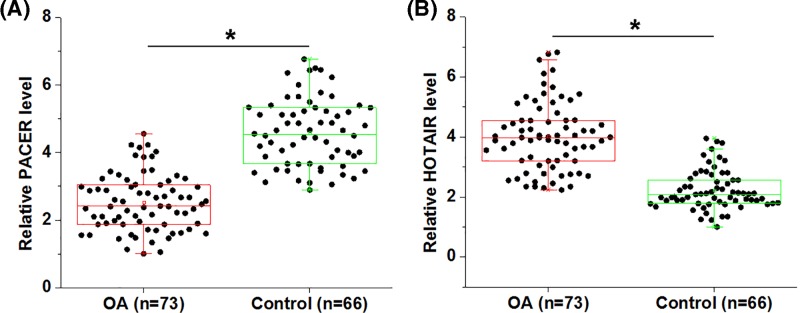
Expression levels of PACER and HOTAIR were altered in OA RT-qPCR results revealed that PACER was down-regulated (**A**), while HOTAIR was up-regulated (**B**) in OA patients compared with healthy controls (*, *P*<0.05).

PACER and HOTAIR in plasma of both OA patients and healthy controls were detected by performing qPCR and compared between unpaired *t-*test. It was observed that plasma levels of PACER were significantly lower in OA patients than in healthy controls (Figure 1A, *P*<0.05). In contrast, plasma levels of HOTAIR were significantly higher in OA patients than in healthy controls (Figure 1B, *P*<0.05).

### Altered plasma levels of PACER and HOTAIR distinguished OA patients from healthy controls

**Figure 2:**
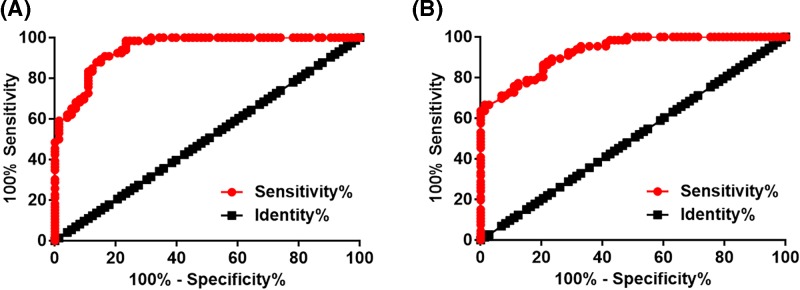
Altered plasma levels of PACER and HOTAIR distingshed OA patients from healthy controls ROC curve analysis showed that altered plasma levels of PACER (**A**) and HOTAIR (**B**) distinguished OA patients from healthy controls.

ROC curve analysis was performed according to aforementioned methods. Area under the curve (AUC) of the use of plasma PACER in the diagnosis of OA was 0.95, with standard error of 0.017 and 95% confidence interval of 0.91–0.98 (Figure 2A). AUC of the use of plasma HOTAIR in the diagnosis of OA was 0.90 (Figure 2B), with standard error of 0.018 and 95% confidence interval of 0.90–0.98.

### PACER and HOTAIR were inversely correlated

**Figure 3:**
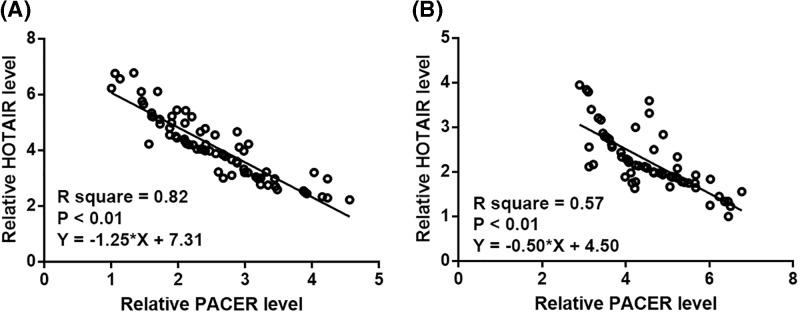
Plasma levels of PACER and HOTAIR were inversely correlated Linear regression analysis revealed that PACER and HOTAIR were significantly and inversely correlated in both OA patients (**A**) and healthy controls (**B**).

Correlations between expression levels of PACER and HOTAIR were analyzed by linear regression. A significant and inverse correlation between PACER and HOTAIR was found among OA patients (Figure 3A). In addition, plasma levels of PACER and HOTAIR were also inversely and significantly correlated in healthy controls (Figure 3B).

### PACER is an upstream inhibitor of HOTAIR in cells of CHON-001 cell line

**Figure 4:**
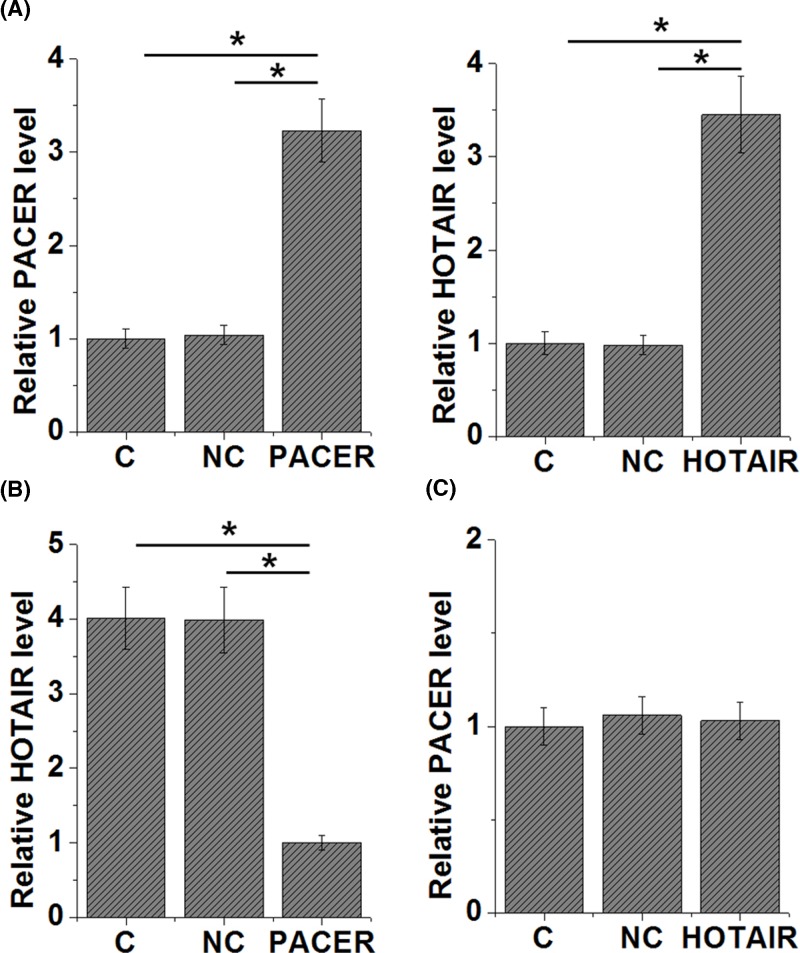
PACER is an upstream inhibitor of HOTAIR in cells of CHON-001 cell line Overexpression of PACER and HOTAIR was reached at 24 h after transfection (**A**) Compared with C and NC groups, PACER overexpression mediated the down-regulation of HOTAIR (**B**), while HOTAIR overexpression did not significantly affect PACER (**C**), (*, *P*<0.05).

The significant and inverse correlation between PACER and HOTAIR indicated the potential existence of interactions between them. PACER and HOTAIR expression vectors were transfected into cells of CHON-001 cell line to further investigate the interaction between PACER and HOTAIR. Overexpression of PACER and HOTAIR was reached at 24 h after transfection (Figure 4A, *P*<0.05). Comparing to C and NC groups, cells with PACER overexpression showed significantly down-regulated HOTAIR expression (Figure 4B, *P*<0.05). In contrast, cells with HOTAIR overexpression showed no significantly altered expression level of PACER (Figure 4C).

### PACER inhibited the apoptosis of cells of CHON-001 cell line through HOTAIR

**Figure 5:**
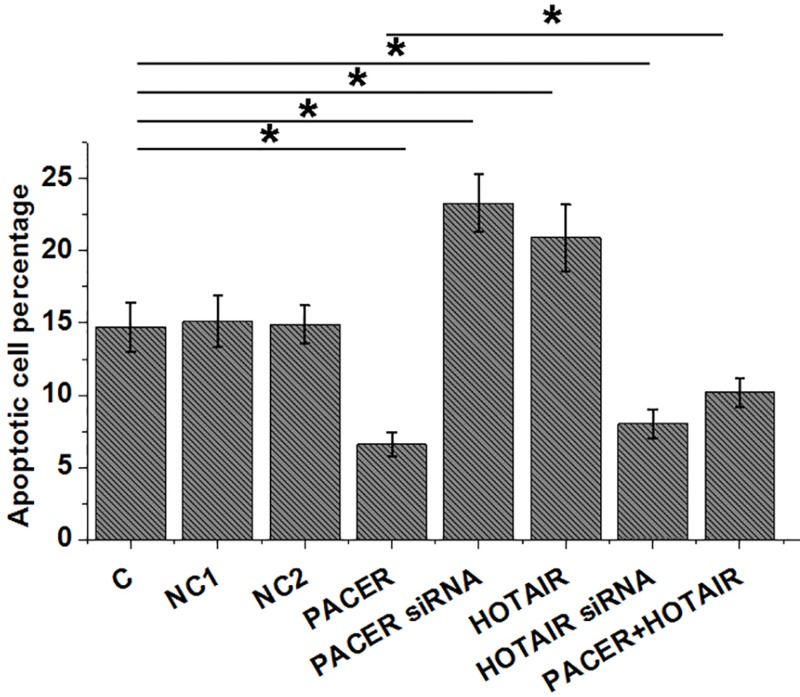
PACER inhibited the apoptosis of cells of CHON-001 cell line through HOTAIR Compared with C and NC groups, PACER overexpression led to inhibited, while HOTAIR overexpression led to promoted apoptosis of chondrocyte. PACER overexpression led to inhibited, while HOTAIR overexpression led to promoted apoptosis of chondrocyte (Figure 5, *P*<0.05). In addition, HOTAIR overexpression attenuated the effects of PACER overexpression on cell apoptosis. NC1, empty vector transfection; NC2, negative control siRNA transfection; *, *P*<0.05.

Comparing to C and NC groups, PACER overexpression led to inhibited, while HOTAIR overexpression led to promoted apoptosis of chondrocyte (Figure 5, *P*<0.05). Moreover, PACER siRNA silencing led to promoted, while HOTAIR siRNA silencing led to inhibited apoptosis of chondrocyte (Figure 5, *P*<0.05). In addition, comparing to cells with PACER overexpression, cells with both PACER and HOTAIR overexpression showed significantly promoted cell apoptosis (Figure 5, *P*<0.05).

## Discussion

The present study mainly explored the involvement of PACER in OA. Our data support the hypothesis that PACER overexpression may improve OA by inhibiting the apoptosis of chondrocyte through the down-regulation of lncRNA HOTAIR, which promotes OA [11].

HOTAIR is a well-characterized oncogenic lncRNA in cancer biology [13]. HOTAIR participate in cancer development and progression mainly by regulating chromatin dynamics and cancer cell behaviors [13]. It is generally believed that HOTAIR inhibits cancer cell apoptosis to promote disease progression [14,15]. In contrast, overexpression of HOTAIR in OA mediates the apoptosis of chondrocytes [13]. Therefore, HOTAIR may play opposite roles in regulating cancer behaviors in different pathological processes. Consistent with previous studies, our study also observed the up-regulation of HOTAIR in OA patients compared with healthy controls. In addition, overexpression of HOTAIR led to the promoted apoptosis of chondrocytes. Our data further confirmed the involvement of HOTAIR in OA.

A recent study reported that inflammatory response in chondrocytes of OA patients is likely related to certain differentially expressed lncRNAs, such as PACER [12]. Our preliminary RNA-seq experiment also revealed the altered expression of PACER in OA patients (data not shown). Interestingly, the present study also showed that overexpression of PACER mediated the inhibited apoptosis of chondrocytes. Therefore, overexpression of PACER may improve OA. However, more experiments are needed to further confirm our hypothesis.

LncRNAs are usually expression in specific cell types or during specific developmental stages to regulate local gene expression [16,17]. However, lncRNAs may also enter blood to systemically regulate gene expression [18]. In the present study, we detected PACER and HOTAIR in plasma of all OA patients and healthy controls, indicating that PACER and HOTAIR may serve as systemic gene expression regulation molecules. Our preliminary RNA-seq experiment also revealed the inverse correlation between PACER and HOTAIR in OA patients (data not shown), indicating the existence of interactions between PACER and HOTAIR. The present study showed that PACER is likely an upstream inhibitor of HOTAIR in chondrocytes. However, molecular mechanism of this interaction is unknown. However, the interaction between PACER and HOTAIR characterized in the present study may guide the studies on the function of HOTAIR in other diseases, such as cancer.

In conclusion, PACER was down-regulated and HOTAIR was up-regulated in OA. PACER overexpression may improve OA by inhibiting the apoptosis of chondrocyte through the down-regulation of lncRNA HOTAIR.

## Abbreviations

AUC: area under the curve
C: control
IncRNA: long non-coding RNA
NC: negative control
OA: osteoarthritis
